# Screening of Asymptomatic Healthcare Workers for SARS-COV-2 for Occult Infections: A Cross-Sectional Study

**DOI:** 10.7759/cureus.19341

**Published:** 2021-11-07

**Authors:** Lakshmi J Tadi, Srinivasa Rao Chunchu, Srinivas M, Saranya Mallamgunta, Ushasree Ravula, Ariyanachi K, Chennakesavulu Dara, Triven Sagar Sandepogu

**Affiliations:** 1 Microbiology, All India Institute of Medical Sciences, Bibinagar, Hyderabad, IND; 2 Transfusion Medicine, ESIC Medical College and Hospital, Hyderabad, IND; 3 Pediatric Surgery, ESIC Medical College and Hospital, Hyderabad, IND; 4 Microbiology, ESIC Medical College and Hospital, Hyderabad, IND; 5 Anatomy, All India Institute of Medical Sciences, Bibinagar, Hyderabad, IND; 6 Medicine, ESIC Medical College and Hospital, Hyderabad, IND

**Keywords:** abo and rh blood groups, sars-cov-2-igg antibodies, seroprevalence, health care workers, sars-cov-2

## Abstract

Introduction

The SARS-CoV-2 illness (COVID-19) has spread around the world, primarily through person-to-person transmission, and is a serious public health concern. Based on the severity of illness symptoms, SARS-CoV-2 infection can be classified as either apparent or occult. To date, real-time reverse transcription polymerase chain reaction (RT-PCR) on respiratory specimens, particularly nasopharyngeal and oropharyngeal swabs, or nasopharyngeal wash or aspirate, has been the gold standard for the identification of COVID-19. A negative RT-PCR does not necessarily rule out SARS-CoV-2 infection. Occult COVID-19 infections could least be identified with RT-PCR.

Aims and objectives

To assess the prevalence of possible occult COVID-19 infection in healthcare personnel by RT-PCR and serology testing for SARS-CoV-2 virus.

Methods

A cross-sectional study was conducted on health care workers at a tertiary care hospital in South India during the period from October 2020 to January 2021. None of the study participants were vaccinated against COVID-19 during the study period. Nasopharyngeal swabs collected for RT-PCR were tested using Cobas 480 platform (Roche, Basel, Switzerland). Peripheral blood venous sampling was performed to collect EDTA (ethylenediaminetetraacetic acid) and plain samples. SARS-CoV-2 IgG antibodies against spike proteins were estimated using ECI Vitros platform (Ortho Clinical Diagnostics, Raritan, USA).

Results

The mean age of study participants was 34.78 years (SD±9.51) with an age range of 19-69 years. The study participants were stratified into age groups of 19-25 years, 26-40 years, 41-60 years, and above 60 years, gender, ABO and Rh blood groups, and occupational and further based on their area of work as Covid and Non-Covid for the purpose of statistical analysis. Total 190 samples from healthcare workers (HCWs) were tested for RT-PCR using nasopharyngeal swabs collected at the time of enrolment into the study, and all the 190 samples tested negative for RT-PCR. Among 190 HCW samples screened for SARS-CoV-2-IgG antibodies, 48 (25.3%) were found reactive for IgG antibodies while 142 (74.7%) were found non-reactive.

Conclusion

Our study findings suggested that using RT-PCR testing, which may only identify those with a prolonged viral shedding period and minimum viral loads, the proportion of asymptomatic/occult infections could be underestimated.

## Introduction

The SARS-CoV-2 illness (COVID-19) has spread around the world, primarily through person-to-person transmission, and is a serious public health concern. Recent research has progressively described the epidemiologic and clinical characteristics of symptomatic COVID-19 patients, but the asymptomatic proportion of SARS-CoV-2 infected individuals remains mostly unknown [1]

Based on the severity of illness symptoms, SARS-CoV-2 infection can be classified as either apparent or occult. Clinical signs and biochemical alterations were frequently absent or modest in occult infections, which could only be discovered through immunological testing. Adequate testing of such apparent and occult COVID-19 infection is of utmost importance, which allows identification of people who might need treatment, or who need to isolate themselves to prevent the spread of infection. Correctly identifying people who have had COVID-19 is critical for determining disease dissemination, evaluating the effectiveness of public health interventions (such as isolation), and maybe identifying those with immunity.

Numerous medical personnel have been fighting on the frontlines since the outbreak of new coronavirus (SARS-CoV-2)-infected pneumonia (COVID-19). However, several recent investigations have overlooked the risk of hidden and occult infection in healthcare personnel and attention is focused only on the patients. Occult COVID-19 infections could seed new outbreaks and it is high time to estimate the proportion of people with mild or no symptoms who could spread the pathogens to others. And by the working nature, occult COVID-19 infections could be assumed to be prevalent among healthcare personnel [2]

To date, real-time reverse transcription polymerase chain reaction (RT-PCR) on respiratory specimens, particularly nasopharyngeal and oropharyngeal swabs, or nasopharyngeal wash or aspirate, has been the gold standard for the identification of COVID-19. Nevertheless, more and more evidence is emerging regarding its lack of adequate sensitivity, questioning whether the current recommendations on COVID-19 diagnosis guarantee an adequate level of safety and effectiveness in the fight against the growing contagion [3,4,5]. A negative RT-PCR does not necessarily rule out SARS-CoV-2 infection. Occult COVID-19 infections could least be identified with RT-PCR. This could be attributed to the low amount of SARS-CoV-2 virus concentration [6]. Serology tests to detect antibodies to SARS-CoV-2 when combined with RT-PCR are better alternatives to identify earlier SARS-CoV-2 apparent and occult infections and may also be used to confirm the existence of current occult COVID-19 infection.

## Materials and methods

Study setting

A cross-sectional study was conducted at a tertiary care hospital in South India from October 2020 to January 2021. Ethical clearance was obtained from Institutional Ethics Committee.

Study participants

The study included various segments of healthcare workers of the Institute working in various departments classified as COVID and Non-COVID areas of work. None of the study participants were vaccinated against COVID-19 during the study period.

Inclusion criteria

Healthcare workers with no prior COVID-19 RT-PCR positive diagnosis were included, after explaining the nature of study and informed consent was obtained. Data like demographic details, area of work, history of exposure to COVID-19 at work and home, use of personal protective equipment (PPE) at the area of work were captured using study proforma during enrollment into the study.

Exclusion criteria

Healthcare workers with a previous history of COVID-19 RT-PCR positive diagnosis were not included in the study.

Nasopharyngeal swabs collected for RT-PCR were tested using Cobas 480 platform (Roche, Basel, Switzerland). Peripheral blood venous sampling was performed to collect EDTA and plain samples. Blood grouping was performed using the Column agglutination technique (Griffols, Barcelona, Spain). SARS-CoV-2 IgG antibodies against spike proteins were estimated using the ECI Vitros platform (Ortho Clinical Diagnostics, Raritan, USA).

Data analysis

The study participants were stratified based on age, gender, occupational groups as doctors, paramedical, housekeeping, and allied and administrative staff; area of work as COVID and non-COVID; ABO and Rh blood groups. The subject samples testing positive for the presence of SARS-CoV-2 antibodies with negative RT-PCR test and no previous history of COVID-19 diagnosis and/or symptoms were considered to have occult infections.

Statistical analysis

The data was analyzed using SPSS 26.0 (IBM Corp, Armonk, USA) and Microsoft Excel software (Microsoft, Redmond, USA). Chi-square test was used to find the association between variables. P-value of <0.05 was considered to be statistically significant. All tests were tallied with a Confidence Interval of 95%.

## Results

Demographic details of study participants

The mean age of study participants was 34.78 years (SD ± 9.51) with age range of 19-69 years. The study participants were stratified into age groups of 19-25 years, 26-40 years, 41-60 years, and above 60 years, gender, ABO and Rh blood groups, occupational and further based on their area of work as COVID and non-COVID for the purpose of statistical analysis (Table 1).

**Table 1:** Demographic distribution of healthcare workers

Study Participants n=190	Total n (%)	Covid Area n=102 (53.7%)	Non-Covid Area n=88 (46.3%)	Pearson Chi-square, P-value
Age, Mean	34.78 years	33.77 years	35.9 years	-
Age Groups				
19-25 years	26 (13.7%)	12 (11.7%)	14 (15.9%)	2.892, 0.409
26-40 years	120 (63.2%)	70 (68.6%)	50 (56.8%)	
41-60 years	38 (20%)	17 (16.6%)	21 (23.8%)	
>60 years	06 (3.2%)	03 (2.9%)	03 (3.4%)	
Gender				
Male	101 (53.2%)	59 (57.8%)	42 (47.7%)	1.941, 0.164
Female	89 (46.8%)	43 (42.1%)	46 (52.3%)	
ABO Blood Group				
A Group	47 (24.7%)	25 (24.5%)	22 (25%)	1.444, 0.695
AB Group	05 (2.6%)	04 (3.9%)	01 (1.1%)	
B Group	58 (30.5%)	31 (30.3%)	27 (30.7%)	
O Group	80 (42.1%)	42 (41.1%)	38 (43.1%)	
RH D Group				
RH D Positive	182 (95.8%)	97 (95.1%)	85 (96.6%)	0.261 0.445
RH D Negative	8 (4.2%)	5 (4.9%)	3 (3.4%)	
Occupational Groups of Health Care Workers				
Doctors	48 (25.3%)	26 (25.4%)	22 (25%)	-
Paramedical Staff	68 (35.8%)	41 (40.1%)	27 (30.6%)	
Housekeeping and Allied	58 (30.5%)	35 (34.3%)	23 (26.1%)	
Administrative Staff	16 (8.4%)	0	16 (18.1%)	

SARS-CoV-2 screening

Antigen Test - RT-PCR

Total 190 samples from healthcare workers (HCWs) were tested for RT-PCR using nasopharyngeal swabs collected at the time of enrollment into the study and all the 190 samples tested negative for RT-PCR.

Antibody Test - SARS-CoV-2-IgG

Among 190 HCWs samples screened for SARS-CoV-2-IgG antibodies, 48 (25.3%) were found reactive for IgG antibodies while 142 (74.7%) were found non-reactive.

Age groups vs serological assay results

Among the 48 HCWs with seropositivity, the majority (58.3%) belonged to the 26-40 years age group, followed by 41-60 years (25%), 19-25 years (14.5%), and above 60 age group (2.08%), although the difference across the age groups was not statistically significant (p=0.726) (Table 2).

**Table 2:** Distribution based on age group vs serological assay results Pearson Chi-square = 1.312, p-value = 0.726

Age group	No. of study participants n=190	COVID-19 IgG reactive n=48 (25.3%)	COVID-19 IgG Non-reactive n=142 (74.7%)
19-25 years	26 (13.7%)	7 (14.5%)	19 (13.3%)
26-40 years	120 (63.2%)	28 (58.3%)	92 (64.8%)
41-60 years	38 (20%)	12 (25%)	26 (18.3%)
>60 years	6 (3.2%)	1 (2.08%)	5 (3.5%)

Gender vs serological assay results

Among 48 HCWs with seropositivity, 45.8% were males and 54.2% were females and no statistical significance was found in relation to gender (p=0.239) (Table 3).

**Table 3:** Distribution based on gender vs serological assay results Pearson Chi-square = 1.384, p-value = 0.239

Gender	No. of study participants n=190	COVID-19 IgG reactive n=48 (25.3%)	COVID-19 IgG Non-reactive n=142 (74.7%)
Male	101 (53.2%)	22 (45.8%)	79 (55.6%)
Female	89 (46.8%)	26 (54.2%)	63 (44.4%)

Blood group vs serological assay results

Among the 48 HCWs with seropositivity, 35.4% had the blood group B, followed by A (31.2%), O (29.1%), and AB (4.1%), and the distribution across ABO and Rh blood groups were not statistically significant (Tables 4, 5).

**Table 4:** Distribution based on ABO blood group vs serological assay Pearson Chi-square = 4.733, p-value = 0.192

ABO blood group	No. of study participants n=190	COVID-19 IgG reactive n=48 (25.3%)	COVID-19 IgG Non-reactive n=142 (74.7%)
A Group	47 (24.7%)	15 (31.2%)	32 (22.5%)
AB Group	5 (2.6%)	2 (4.1%)	3 (2.1%)
B Group	58 (30.5%)	17 (35.4%)	41 (28.9%)
O Group	80 (42.1%)	14 (29.1%)	66 (46.47%)

**Table 5:** Distribution based on Rh D blood group vs serological assay Pearson Chi-square = 0.662, p-value = 0.326

RH D Group	No. of study participants n=190	COVID-19 IgG reactive n=48 (25.3%)	COVID-19 IgG non-reactive n=142 (74.7%)
RH D Positive	182 (95.8%)	45 (93.7%)	137 (96.4%)
RH D Negative	8 (4.2%)	3 (6.2%)	5 (3.5%)

Area of work vs serological assay results

Among 190 HCWs screened, 52.1% of HCWs working in Non-COVID areas and 47.9% working in COVID areas had SARS-CoV-2-IgG antibodies. There was no significant difference in the presence of antibodies among the healthcare workers with respect to the area of work (p=0.354) (Table 6).

**Table 6:** Distribution based on area of work vs serological assay Pearson Chi-square = 0.859, p-value = 0.354

Area of work	No. of study participants n=190	COVID-19 IgG reactive n=48 (25.3%)	COVID-19 IgG Non-reactive n=142 (74.7%)
Covid Area	102 (53.6%)	23 (47.9%)	79 (55.6%)
Non-Covid Area	88 (46.3%)	25 (52.1%)	83 (58.4%)

Occupation vs serological assay results

Serological assay results in different occupations among the healthcare workers results showed the highest seropositivity among the housekeeping and allied (35.4%), followed by paramedical (31.2%), doctors (20.8%), and administrative staff (12.5%), although no statistical significance was found (Table 7).

**Table 7:** Distribution based on occupation vs serological assay Pearson Chi-square = 2.641, p-value = 0.450

Occupation	No. of study participants n=190	COVID-19 IgG reactive n=48 (25.3%)	COVID-19 IgG Non-reactive n=142 (74.7%)
Doctors	48 (25.3%)	10 (20.8%)	38 (26.7%)
Paramedical Staff	68 (35.8%)	15 (31.2%)	53 (38.7%)
Housekeeping & Allied	58 (30.5%)	17 (35.4%)	41 (28.8%)
Administrative Staff	16 (8.4%)	6 (12.5%)	10 (7%)

## Discussion

SARS-CoV-2 infection can cause a variety of symptoms, ranging from asymptomatic sickness to multisystem organ failure and even death. However, most individuals have few or no symptoms, making it difficult to prevent disease spread. So researchers are scrambling to solve a key epidemiological puzzle: how many infected people show mild or no symptoms and may be passing the virus on to others? Despite the fact that all healthcare workers receive protective training before starting work, several studies on healthcare workers have reported COVID-19 as a highly contagious disease that could be apparent or occult. This could be attributed to a poor understanding of the epidemiology of the SARS-CoV-2 virus. The current study was aimed to know the prevalence of occult COVID-19 infections among healthcare workers in a tertiary care hospital. During the study period, the institute was providing designated COVID and Non-COVID services with dedicated staff.

In our study, 190 healthcare personnel of the Institute, working in COVID and Non-COVID areas were screened for SARS-CoV-2 infection by real-time RT-PCR and serological assays. None of the study participants were vaccinated against COVID-19 during the study period. All tested negative for RT-PCR but 48 (25%) HCWs were found seropositive for SARS-CoV-2 IgG antibodies. None of the HCWs reported any clinical symptoms pertaining to COVID-19 nor had any prior COVID-19 RT-PCR positive diagnosis. These 48 HCWs can be considered to have had occult infections. Similar to our study findings, the possibility of occult infections was speculated among hospital staff in addition to apparent infections by Li et al. and other studies [2,7,8]. However, more research into the infectivity of these occult illnesses based on exposure time in working areas, type of PPE, and their outcomes or presentation is needed.

The study reported the majority of seropositivity among the 26-40 years age group with slight female preponderance when compared across the age groups, although no statistical significance was found. No other study had previously evaluated the gender differences in occult COVID-19 infections. However, a few studies like Syangtan et al. and Yang et al. analyzed the gender differences in asymptomatic COVID-19 infections and reported a higher frequency of asymptomatic SARS-CoV-2 infection in females than males correlating with our study findings [9,10].

Our study is the first to report the prevalence of occult COVID-19 infections among HCWs with blood groups. Although several studies have interpreted the relationship between blood types and confirmed COVID-19 symptomatic cases [11-14], no study has elicited the ABO Rh blood group status among the occult COVID-19 infections. Among ABO blood groups, the incidence of occult infections was found to be more among the HCWs with B blood group followed by A, O, and AB. However, no statistical significance was elicited among ABO and Rh Blood groups with serological reactivity. Further, the incidence of infection with blood types may vary from population to population.

With regard to the area of work, 102 HCWs enrolled were working in COVID areas while 88 were working in Non-COVID areas; 22.5% Sero-positivity was found in HCWs working in the COVID area (23/102), while seropositivity of 28.4% was found in HCWs working in the Non-COVID area (25/88). Although the seropositivity rate appears to be slightly higher in the Non-COVID area than the COVID area, the difference is not statistically significant. This can be attributed to the fact that there was the use of full personal protective equipment (PPE) by HCWs in COVID areas in comparison to those working in Non-COVID areas with minimal PPE. No study has evaluated the prevalence of occult infections between various working groups but studies have evaluated the incidence of symptomatic cases between different workgroups.

Serological assay results in different occupations among the health care workers results showed the highest seropositivity among the housekeeping and allied (35.4%), followed by paramedical (31.2%), doctors (20.8%), and administrative staff (12.5%), although no statistical significance was found. Other studies stated work-related transmission to be considerable in early COVID-19 outbreaks, and the elevated risk of infection was not limited to HCW [15]. Our study also showed that no specific occupation was at higher risk of occult infections. Yet the use of full or minimal personal protective equipment at respective work areas among health care workers can be correlated to the seroprevalence results obtained in our study [16]. Implementing preventive/surveillance strategies for high-risk working populations should be considered based on seroprevalence studies in work areas during the pandemic or epidemics.

Limitations

The exposure time and atypical presentation of symptoms could not be evaluated in our study. Study population size was small and further a larger cohort or multi-centric studies will help to understand the characteristics of COVID-19 occult infections among healthcare workers.

## Conclusions

In epidemic or pandemic prevention, asymptomatic individuals are often overlooked. Our study findings suggested that using RT-PCR testing, which may only identify those with a prolonged viral shedding period and minimum viral loads, the proportion of asymptomatic/occult infections could be underestimated. Serological tests in addition to RT-PCR could be a more reliable way to assess the fraction of occult COVID-19 infections. Finally, more research is needed to confirm that SARS-CoV-2 occult infections are possible virus carriers based on exposure and outcomes.
